# Methanol (80%) leaf extract of *Otostegia integrifolia* Benth (Lamiaceae) lowers blood pressure in rats through interference with calcium conductance

**DOI:** 10.1186/s12906-021-03222-4

**Published:** 2021-02-04

**Authors:** Abel Degu, Abiy Abebe, Ephrem Engidawork

**Affiliations:** 1 Department of Pharmacology and Clinical Pharmacy, School of Pharmacy, College of Health Sciences, Addis Ababa University, P.O. Box 1176, Addis Ababa, Ethiopia; 2 Directorate of Traditional and Modern Medicine Research, Ethiopian Public Health Institute, P.O. Box 1242, Addis Ababa, Ethiopia

**Keywords:** Antihypertensive, Calcium channel blockade, Fructose induced hypertension, *Otostegia integrifolia*, Vasorelaxation

## Abstract

**Background:**

*Otostegia integrifolia* Benth. (Lamiaceae) leaves are used to treat hypertension in Ethiopian folk medicine. However, the claim has so far not been investigated scientifically. Thus, the objective of this study was to evaluate the antihypertensive activity of 80% methanol leaf extract of *O. integrifolia* in animal model of hypertension and possible underlying mechanisms in isolated rat aorta.

**Methods:**

Antihypertensive effect of various oral doses of the extract (250, 500 and 1000 mg/kg) was determined in fructose-induced hypertensive rats using the non-invasive tail-cuff method. Thoracic aortic strips of rats were isolated and suspended in organ bath, and the vasodepressor effect as well as the possible mechanism (s) of action were studied by means of isometric tension recording experiments ex vivo. Phytochemical analysis was also performed to suggest possible constituents related to the activity.

**Results:**

Blood pressure was significantly lowered in a dose-dependent manner following extract administration, suggesting that the extract possesses antihypertensive activity. The extract also caused a dose-dependent relaxation of aortic strip precontracted with KCl at a concentration of 6.25–125 μg/L, with a maximum relaxation (100%) achieved at a cumulative concentration of 318.75 μg/ml. The relaxation mechanism was found to be independent of muscarinic receptors, prostanoids, histamine receptors, ATP dependent K^+^ channels, sarcoplasmic reticulum stored Ca^2+^ and the endothelium system. The extract shifted the Ca^2+^ concentration-response curve to the right similar to that caused by nifedipine, suggesting that vasorelaxation could possibly be mediated via calcium channel blockade. The extract was found to contain phenolic compounds (164.3 mg/g, expressed as gallic acid equivalents) and flavonoids (125.7 mg/g, expressed as quercetin equivalents).

**Conclusion:**

The findings revealed that the plant is endowed with antihypertensive activity, providing evidence for its traditional use. The effect maybe, at least in part, due to dilation of blood vessels through blockade of Ca^+ 2^ channels mediated by phenolic and flavonoid constituents.

**Supplementary Information:**

The online version contains supplementary material available at 10.1186/s12906-021-03222-4.

## Background

Hypertension is one of the major causes of cardiovascular-related morbidity and mortality in humans [1–4]. Epidemiological studies reported that excess dietary fructose (≥74 g/day) in the form of added sugar is associated with higher blood pressure (BP) values in adults who did not have a history of hypertension [5]. In animals, high-fructose diets are used for decades to generate models of hypertension and insulin resistance [6], through mechanisms involving salt retention, endothelial dysfunction and sympathetic activation [7].

Although the available anti-hypertensive agents have managed to achieve optimal BP in the majority of patients, attaining target BP and reducing the potential for acquiring cardiovascular complications are still far from possible in some group of patients [8]. Medication-related problems are considered as one of the major contributing elements, particularly in elderly patients as well as in patients with comorbid conditions, on poly-pharmacy and resistant hypertension [9]. This may end up in poor adherence and treatment failure [10]. Due to these problems as well as the incessant urge and endeavor of mankind to meet the ongoing demand, there is a need to look for viable alternatives [11]. The best way to start is exploring medicinal plants, as they have been used by many since prehistoric times [12]. Ethiopia is blessed with huge biodiversity and ethnobotanical data, which offer an opportunity to scientifically test active plant constituents for their therapeutic potential [13].

The genus *Otostegia* (Lamiaceae) consists of about 15 species, which are endemic to the northern part of tropical Africa as well as South-western and Central Asia. The genus is known to contain terpenes, flavonoids, and iridoids [14, 15]. Five species of this genus have been reported to occur in the flora of Ethiopia, including *Otostegia integrifloia* Benth [16]. *O. integrifolia* is an erect perennial shrub, much branched and spiny, 1–3 m, and is endemic to Ethiopia, Eritrea, and Yemen [16]. It is commonly known with the vernacular name of “Tunjite” in Amharic, and the morphological characteristics is described elsewhere [17]. Phytochemical investigations indicated that *O. integrifolia* contains prefuranic and furanic labdane diterpenoids such as otostegindiol and preotostegindiol, pentatriacontane, and stigmasterol [16] as well as phenolic compounds, saponins, and flavonoids [18].

Pharmacological investigations revealed that the plant possesses several properties, including antimalarial [16], antimicrobial and antioxidant [18] as well as acaricidal [19] activities. The leaves of *O. integrifolia* have also traditionally been used for treatment of hypertension by the Eritrean [20] and Ethiopian (in Tigray) [13] people for decades. Thus, the purpose of this study was to verify whether the plant of interest possesses the claimed antihypertensive activity using in vivo and ex vivo models.

## Methods

### Plant material

The leaves of *O. integrifolia* were collected from a town called Tulu Dimtu, located in North West Shewa, Oromiya region (geographical coordinates are 9° 41′ 0“ North, 38° 40’ 0” East), about 29 km southeast of Addis Ababa, Ethiopia, in December 2016. Identification & authentication of the plant specimen was done by a taxonomist and a voucher specimen (AD001) was deposited at the National Herbarium, College of Natural and Computational Sciences, Addis Ababa University, for future reference. The plant material was thoroughly washed with tap water to remove dirt and soil. The leaves were then dried at room temperature under shade and powdered using mortar and pestle.

### Experimental animals

Sprague-Dawley rats (250–300 g, 6–8 weeks of age) were obtained from the animal house of School of Pharmacy and animal unit of the Ethiopian Public Health Institute, Addis Ababa, Ethiopia. They were provided with standard pellet and water *ad libtum* under a controlled environment (12 h light– dark cycle and temperature of 23–25 °C). The animals were acclimatized for a week with the tail cuff instrument and the environment. The care and handling of animals were in line with international guidelines [21] and the protocol was approved by IRB of the School of Pharmacy (Reference no. ERB/SOP/120/11/2017).

### Extraction

Five hundred gram of air-dried and powdered plant material was extracted with 80% methanol by cold maceration, at room temperature, for three consecutive days. The procedure was repeated twice by adding another fresh solvent to the marc. The resulting liquid extract was combined and filtered using a Whatman no. 1 filter paper. The filtrates were combined and concentrated using rotavapor (Buchilabortechnik AG, Switzerland) at 40 °C under reduced pressure. The concentrated extract was then freeze-dried using a lyophilizer (Heto Power Dry LL3000 freeze-dryer, USA). A yellowish brown, particularly of apricot type, hygroscopic shiny powder with percentage yield of 16.6% (w/w) was obtained. The resulting extract was then transferred into a vial and kept in a refrigerator (4 °C) until further use.

### Phytochemical analysis

The 80% methanol leaf extract of *O. integrifolia* was screened for the possible presence of secondary metabolites, including alkaloids, tannins, flavonoids, terpenoids, saponins, and phenols using qualitative phytochemical screening procedures as described elsewhere [22].

#### Determination of total phenol content

The total phenol content of the extract was determined using Folin Ciocalteu reagent with minor modification [23]. Briefly, one ml of aliquots of gallic acid (standard) (100, 50, 25, 12.5, and 6.25 μg/mL) was mixed and shaken with 5 ml of distilled water and 0.5 ml of Folin Ciocalteu’s reagent. After 5 min, 1.5 ml of 20% sodium carbonate was added and the volume was made up to 10 ml with distilled water. The absorbance was then measured at 750 nm after 2 h of incubation at room temperature. The same procedure was also followed for the extract (100 μg/mL) as well as the blank. All measurements were performed in triplicate and the average value was taken. A calibration curve was plotted (Supplementary Figure 1) to determine the concentration of total phenols and the result was expressed as gallic acid equivalent (mgGAE/g).

#### Determination of total flavonoid content

The total flavonoid content of the extract was determined by Aluminum chloride method as described elsewhere [23], with minor modification. Briefly, five serially diluted quercetin (standard) solutions with concentrations of 0.0625, 0.125, 0.25, 0.5 and 1 mg/ml were prepared in methanol. One ml of each of the concentrations was mixed with 0.3 ml of 5% sodium nitrate and allowed to stand for 5 min. Similar amount of 10% Aluminum chloride solution was added and allowed to stand for 5 min, after which 2 ml solution of 1 M sodium hydroxide was added sequentially followed by addition of distilled water up to the marc. The mixture of each solution was incubated for 30 min at room temperature. The absorbance of this reaction mixture was recorded at 510 nm using a UV spectrophotometer. The same procedure was repeated with the extract (1 mg/ml) and the blank solutions. All measurements were performed in triplicate. A calibration curve was plotted (Supplementary Figure 2) and the results were expressed as quercetin equivalent (mg QE/g).

### Grouping and dosing of animals

A total of 36 Male rats were used for the in vivo experiment. Rats were randomly assigned into six groups, 6 rats per group. The group included normal control rats (NCR) that received distilled water, negative control rats that received 66% w/v D-Fructose (DF66), positive control rats treated with Captopril 20 mg/kg/day (CAP20) +  66% w/v D-Fructose, and extract group rats treated with 250 mg/kg (OI250), 500 mg/kg (OI500), or 1000 mg/kg (OI1000) of the extract plus 66% w/v D-Fructose. All doses were administered daily per oral route for fifteen consecutive days and the maximum volume administered was 20 ml/kg. Dose selection was made based on pilot experiments as well as acute oral toxicity study performed on the plant [16].

### Induction of experimental hypertension

Hypertension was induced using the dietary method employing D-fructose 66% w/v. For this purpose, all groups of animals, except the NCR, received fructose daily through drinking water ad libitum for a total of 15 days. Consumption of fructose leads to development of hypertension through activation of the sympathetic nervous system, increased salt retention and enhanced renin-angiotensin-aldosterone system (RAAS) [24, 25].

### Blood pressure measurement

**Fig. 1:**
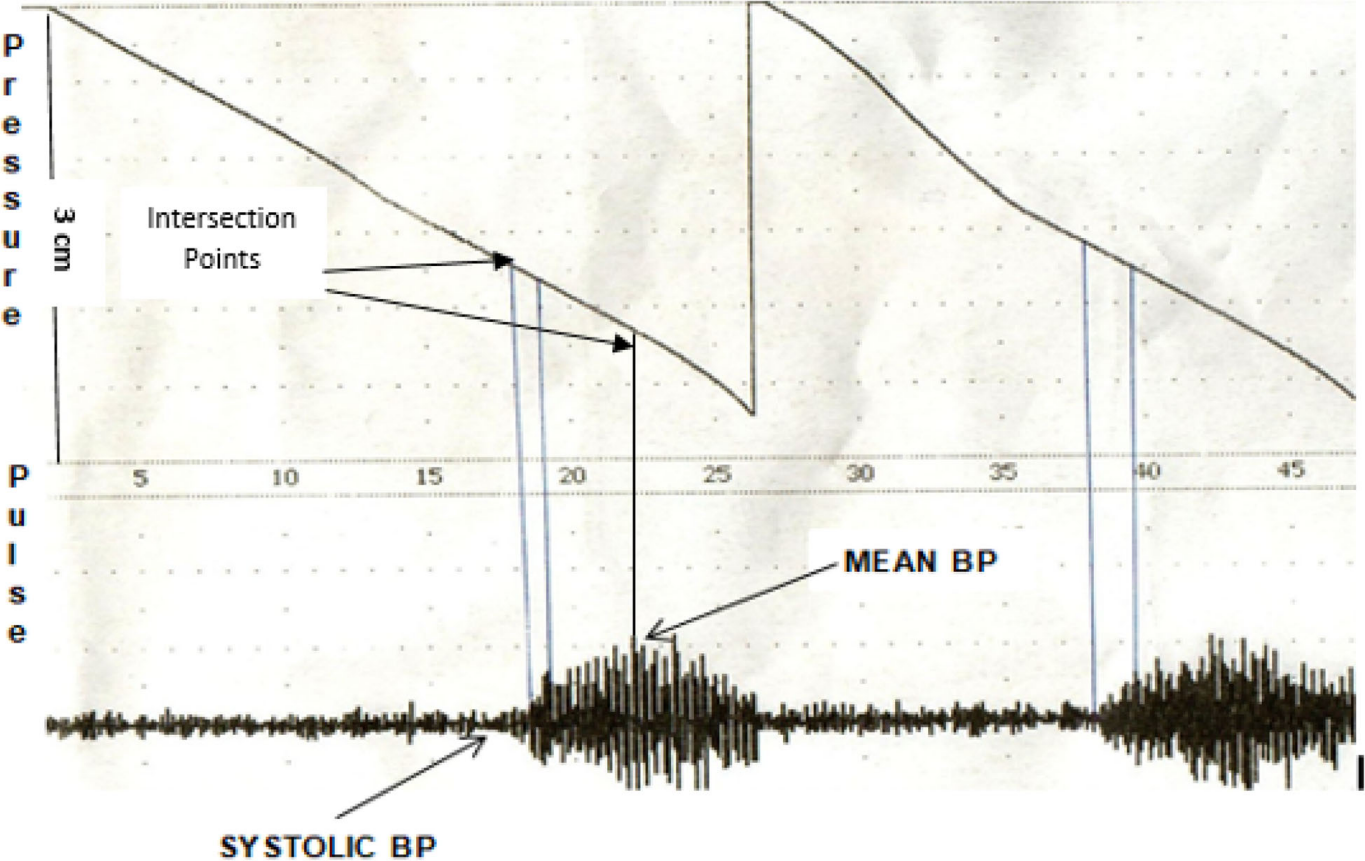
A representative tracing of rat blood pressure measurement showing the pressure of tail cuff and pulse: the total length from the base of pulse channel up to the pressure channel is 3 cm, which is equivalent to 300 mmHg of pressure. The lowest point (base) and highest peak from the pulse channel was extrapolated to the pressure channel. The intersection in the pressure channel was marked then the length from the intersection up to the respective points was measured and calculated to give the corresponding blood pressure reading for systolic and mean arterial blood pressure, respectively. The diastolic blood pressure was calculated using this equation DBP = (3MAP – SBP)/2

BP was measured from the tail of rats using a non-invasive BP monitoring apparatus (Model 179, IITC Inc., USA). For testing, the animals were placed in a pre-warmed holder. The appropriate cuff with sensor (photoelectric) was then mounted on the tails and warmed to about 32–34 °C. When the rats were relaxed and became calm, the tail cuff was inflated to a pressure well above the expected systolic BP (SBP) (200 mmHg) and slowly released during which the pulse was recorded by the BP analyzer. The SBP and mean arterial BP (MAP) were read from the pulse tracings (Fig. 1). The diastolic BP (DBP) was calculated from SBP and MAP using the equation: DBP = (3MAP – SBP)/2 [19]. Pulse pressure (PP) was obtained by subtracting DBP from SBP. Measurement was made in triplicate and average values of the triplicates were reported.

### Ex vivo vasorelaxant activity

The experiment was conducted on rat aortic strips according to the method described elsewhere [26]. Ten rats were sacrificed after being anesthetized with intraperitoneal (ip) pentobarbital (50–60 mg/kg) and the descending thoracic aorta was then immediately removed and placed in Krebs-Henseleit solution (composition in mM: 118.2 NaCl, 4.7 KCl, 1.2 MgSO4, 2.5 CaCl_2_.2H2O, 1.3 KH_2_PO_4_, 25 NaHCO_3_ and 11.7 glucose; pH 7.4). Excess fat and connective tissues were trimmed off and cut spirally to make a strip of about 3 mm wide and 4 cm long. The tissue was then kept moistened with Krebs-Henseleit solution during the whole procedure and finally mounted in an organ bath containing 20 ml of the physiological solution at 37 °C and aerated with carbogen (95% of oxygen and 5% of carbon dioxide).

A resting tension of 1 g was applied to the tissue and an equilibrium period of 60–70 min was allowed before addition of any drug or the test extract, during which it was washed every 15 min. Effect of the extract was first determined on the resting baseline of the tissue to see if it had any vasoconstrictor effect. After stabilization, the aorta was contracted with KCl. Once a contraction plateau was achieved, various concentrations of the extract dissolved in distilled water were cumulatively added and tension changes of the tissue were recorded. The effect of the extract on resting tension was tested with isometric sensors and traced using a recorder (Grass model 7E polygraph).

### Evaluation of possible mechanism of vasorelaxation

The possible mechanism (s) of vasorelaxation produced by the extract was partly studied using different agonists and antagonists. To check the involvement of cholinergic, prostanoids (PGI_2_) and NO; the tissue was pre-incubated with 0.1 μM atropine (antimuscarinic), 10 μM indomethacin (non-selective inhibitor of cyclooxygenases), 100 μM L-NAME (NO synthase inhibitor), respectively, for 15 min before adding the test substance. Any involvement of ATP dependent K+ channel and histamine was studied by preincubating the tissue with the respective inhibitors, glibenclamide (10 μM), and diphenhydramine (10 μM) [27].

To investigate the role of endothelium, the procedure was carried out in endothelium-denuded aorta. The endothelium lining of the aortic strips was removed mechanically by gently rubbing the intimal surface of the aortal strip with a moist wooden stick for approximately 30 s. Denudation of endothelium was assessed by determining acetylcholine (0.1–0.3 μM) elicited relaxation in aortic strips pre-contracted with high K^+^ (80 mM). The extract was then tested for its ability to relax the contraction induced by high K^+^. In addition, the role of sarcoplasmic reticulum sequestered calcium was assessed by incubating the tissue with phenylephrine (0.1 μM) [28].

Another series of experiments were performed in order to determine the inhibitory effect of the extract on extracellular Ca^2+^-induced responses. To confirm calcium channel blocking (CCB) activity, concentration-response curves (CRCs) of Ca^2+^ were constructed [28]. For this purpose, the tissue was stabilized in normal Kreb’s solution and then placed in Ca^2+^free Kreb’s solution, containing EDTA (0.1 mM) for 30 min. This solution was further replaced with K^+^ rich (50 mM) and Ca^2+^ free Kreb’s solution, having the following composition (mM) (KCl 50, NaCl 50.58, MgSO_4_ 3.1, NaHCO_3_ 23.8, KH_2_PO_4_ 1.26, glucose 11.1 and EDTA 0.1). Control CRCs of Ca^2+^ were then obtained after an incubation period of 1 h. Following construction of the CRCs, the tissue was then pre-treated with the extract for 50–60 min to assess possible CCB effect. Finally, the Ca^2+^ CRCs were reconstructed in the presence of the highest concentration of the test material. In all experiments, the endothelium was removed by gently rubbing the luminal surface [29].

### Statistical analyses

All results of the in vivo study were expressed as mean + standard error of the mean (SEM). Within group analysis was performed using one-way ANOVA followed by Tuckey’s post-hoc test. The effect of the extract over time was analyzed by Two-way ANOVA followed by Bonferroni test. With regard to the ex vivo studies, the values were expressed as percentage contraction, taking the high K^+^ induced contraction before application of the test extract as 100%. The graphing was performed using Graph Pad Prism software version 7.00 for Windows (Graph Pad Software Inc., San Diego, California, USA). *P*-values of less than 0.05 were considered statistically significant.

## Results

### Phytochemical analysis

Preliminary phytochemical screening of the 80% methanol leaf extract of *O. integrifolia* revealed that the extract contains phenolic compounds, saponins, and flavonoids, while alkaloids, tannins and steroidal compounds were absent. Subsequent analysis showed that the extract indeed contains phenolic compounds and flavonoids, with the total phenol and flavonoid content of 164.3 mg GAE/g and 125.7 mg QE/g, respectively.

### Induction of acute hypertension

A tracing of BP measurement from fructose-induced hypertensive rats is depicted in Fig. 1. Fructose ingestion significantly increased (*p* < 0.001) BP at the different time points (Table 1), where BP measurements were performed. Maximum increase in the three parameters of BP was observed on Day 5, the increase being on average 56, 84, and 71%, for SBP, DBP, and MAP, respectively**.** Whilst the increase for SBP, DBP, MAP and PP on day 10 was 36, 61, 50 and 54%; that of Day 15 was 28, 60, and 43%, respectively.

**Table 1:** Blood pressure changes after induction of hypertension with 66% w/v D-fructose in rats

BP	Group	BP Measurement Period			
		Day 0	Day 5	Day 10	Day 15
**SBP**	**NCR**	112.75 + 3.99	109.50 + 4.31	101.16 + 7.2	111.41 + 0.96
	**DF66**	118.66 + 4.8	185.16 + 6.68***	161 + 9.25 ***	151.58 + 4.02***
**DBP**	**NCR**	69.00 + 6.91	84.00 + 5.49	80.37 + 6.95	78.79 + 3.43
	**DF66**	89.66 + 5.03	164.54 + 6.69***	144.62 + 10.84***	143.58 + 4.02***
**MAP**	**NCR**	83.58 + 3.80	92.50 + 4.92	87.41 + 7.07	89.66 + 2.41
	**DF66**	99.33 + 4.51	171.41 + 6.41***	150.25 + 10.26***	142.91 + 4.88***
	**NCR**	43.75 + 10.12	16.87 + 2.64	33.62 + 5.2	22.00 + 1.95
**PP**	**DF66**	25.50 + 3.05	14.00 + 4.11	18.32 + 1.52*	22.00 + 2.35

Data are expressed as mean ± SEM (*n* = 6); Analysis was performed by one-way ANOVA; *SBP* Systolic blood pressure, *DBP* Diastolic blood pressure, *MAP* Mean arterial pressure, *PP* Pulse pressure, *NCR* Normal control rats without fructose ingestion, *DF66* Rats with 66% fructose ingestion; *p < 0.05; ***p < 0.001.

All BP parameters were found to decrease significantly (*p* < 0.001) between day 5 and 10 in the negative control group. However, no significant decrement was recorded between day 10 and 15 measurements.

### Effect of the extract on hypertensive rats

Daily oral administration of the extract produced a significant antihypertensive activity on the three measurement points. SBP was significantly decreased by all doses of the extract at all time-points compared to DF66 (Table 2).

**Table 2:** The effect of *Otostegia integrifolia* extract on systolic blood pressure in fructose induced hypertensive rat

Group	Systolic BP			
	Day 0	Day 5	Day 10	Day 15
**DF66**	118.66 + 4.8	185.16 + 6.68	161 + 9.25	151.58 + 4.02
**CAP20**	108.41+ 2.45	98.66 + 4.81 b^3^c^3^d^3^e^3^	96.33 + 7.91 b^3^c^1^	93.00 + 5.31 b^3^
**OI250**	116.16+ 3.49	150.66 + 8.79 a^2^	135 + 13.17	109.75 + 11.64 a^3^
**OI500**	121.91+ 4.76	140 + 6.26 a^3^	87.08 + 9.24 a^3^ f^2^	109.33 + 3.08 a^3^
**OI1000**	110.25+ 6.13	142.41 + 3.17a^3^	96.91 + 4.51 a^3^ f^1^	102.91 + 4.17 a^3^

Data are expressed as mean ± SEM (*n* = 6); Analysis was performed by one-way ANOVA; a, DF66 vs OI; b, DF66 vs CAP20; c, OI250 vs CAP20; d, OI500 vs CAP20; e, OI1000 vs CAP20; f, OI250 vs OI500 and OI100; *DF66* Rats with 66% fructose ingestion, *CAP20* Captopril 20 mg/kg, *OI Otostegia integrifolia* at doses of 250, 500 and 1000 mg/kg; 1*p* < 0.05; 2*p* < 0.01; 3*p* < 0.001.

**Fig. 2:**
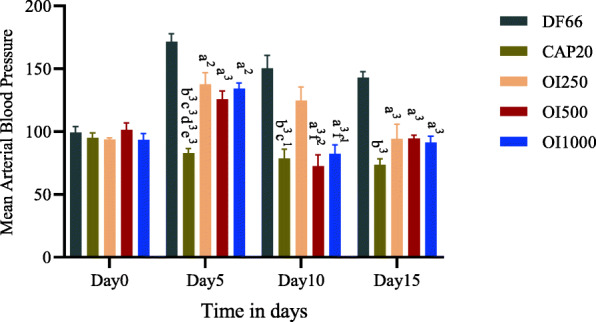
Effect of 80% methanol leaf extract of *Otostegia integrifolia* on mean arterial blood pressure in fructose induced hypertensive rat. Data are expressed as mean ± SEM (*n* = 6); Analysis was performed by one-way ANOVA; **a**, DF66 vs OI; **b**, DF66 vs CAP20; **c**, OI250 vs CAP20; **d**, OI500 vs CAP20; **e**, OI1000 vs CAP20; **f**, OI250 vs OI500 and OI100; DF66, rats with fructose ingestion; CAP20, Captopril 20 mg/kg; OI, *Otostegia integrifolia* at doses of 250, 500 and 1000 mg/kg; 1*p* < 0.05; 2*p* < 0.01; 3*p* < 0.001

**Fig. 3:**
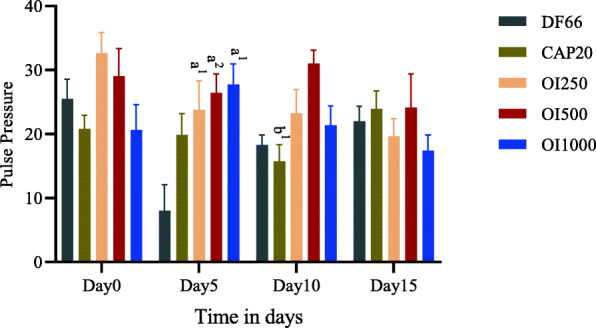
Effect of 80% methanol leaf extract of *Otostegia integrifolia* on pulse pressure in fructose induced hypertensive rat. Data are expressed as mean ± SEM (*n* = 6); Analysis was performed by one-way ANOVA; **a**, DF66 vs OI; **b**, CAP20 vs OI500; DF66, rats with fructose ingestion; CAP20, Captopril 20 mg/kg; OI, *Otostegia integrifolia* at doses of 250, 500 and 1000 mg/kg; 1*p* < 0.05; 2*p* < 0.01; 3*p* < 0.001

DBP also significantly reduced by the extract at all measured days, but the significance was lost at Day 10 with OI250 (Table 3). Daily oral administration of the extract produced a change in MAP that was similar to DBP. OI250, once again, failed to exhibit a decrease in MAP on Day 10 (Fig. 2). Comparison among the different doses of the extract revealed that OI500 (*p* < 0.01) and OI1000 (*p* < 0.05) showed a significantly higher decrease in SBP, DBP, and MAP on day 10 compared to OI250. However, no apparent change in reduction was observed between OI500 and OI1000 at all time-points (Tables 2 and 3, Fig. 2). On the other hand, all doses of the extract lowered PP, however, significant decline (*p* < 0.05) was observed only on day 5 (Fig. 3).

**Table 3:** The effect of *Otostegia integrifolia* extract on diastolic blood pressure in fructose induced hypertensive rat

Group	Diastolic BP			
	Day 0	Day 5	Day 10	Day 15
**DF66**	89.66 + 5.03	164.54 + 6.69	144.62 + 10.84	143.58 + 4.02
**CAP20**	88.54 + 4.86	74.91 + 3.85 b^3^c^3^d^3^e^3^	69.87 + 8.07 b^3^ c^1^	65.25 + 3.94 b^3^
**OI250**	82.54 + 2.43	132.34 + 10.16 a^1^	119.25 + 10.83	86.50 + 11.58a^3^
**OI500**	90.91 + 6.14	118.61 + 6.78 a^3^	65.08 + 9.24 a^3^ f^2^	87.33 + 2.77 a^3^
**OI1000**	86.33 + 5.70	127.75 + 5.37 a^2^	72.79 + 8.00 a^3^ f^1^	85.54 + 5.34 a^3^

Data are expressed as mean ± SEM (*n* = 6); Analysis was performed by one-way ANOVA; a, DF66 vs OI; b, DF66 vs CAP20; c, OI250 vs CAP20; d, OI500 vs CAP20; e, OI1000 vs CAP20; f, OI250 vs OI500 and OI100; *DF66* Rats with fructose ingestion, *CAP20* Captopril 20 mg/kg; OI, *Otostegia integrifolia* at doses of 250, 500 and 1000 mg/kg; 1*p* < 0.05; 2*p* < 0.01; 3*p* < 0.001.

**Fig. 4:**
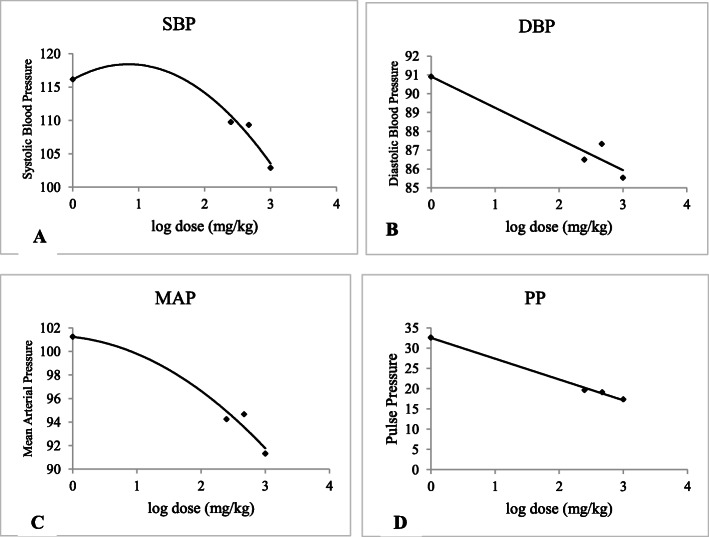
Concentration-response curve of 80% methanol leaf extract of *Otostegia integrifolia* on blood pressure parameters. SBP, Systolic blood pressure; DBP, Diastolic blood pressure; MAP, Mean arterial pressure; PP, Pulse pressure

Overall, the trend over time in SBP, DBP and MAP reduction was significant (*p* < 0.001) between day 5 and 10, but lacked consistency later on between day 10 and day 15 (Table 2). Dose-response curves were constructed and EC_50_ values for the extract were found to be 438.11, 290.68, 328.6, and 279.1 mg for SPB, DBP, MAP, and PP, respectively (Fig. 4).

### Ex vivo studies

#### Vasodepressor activity

**Fig. 5:**
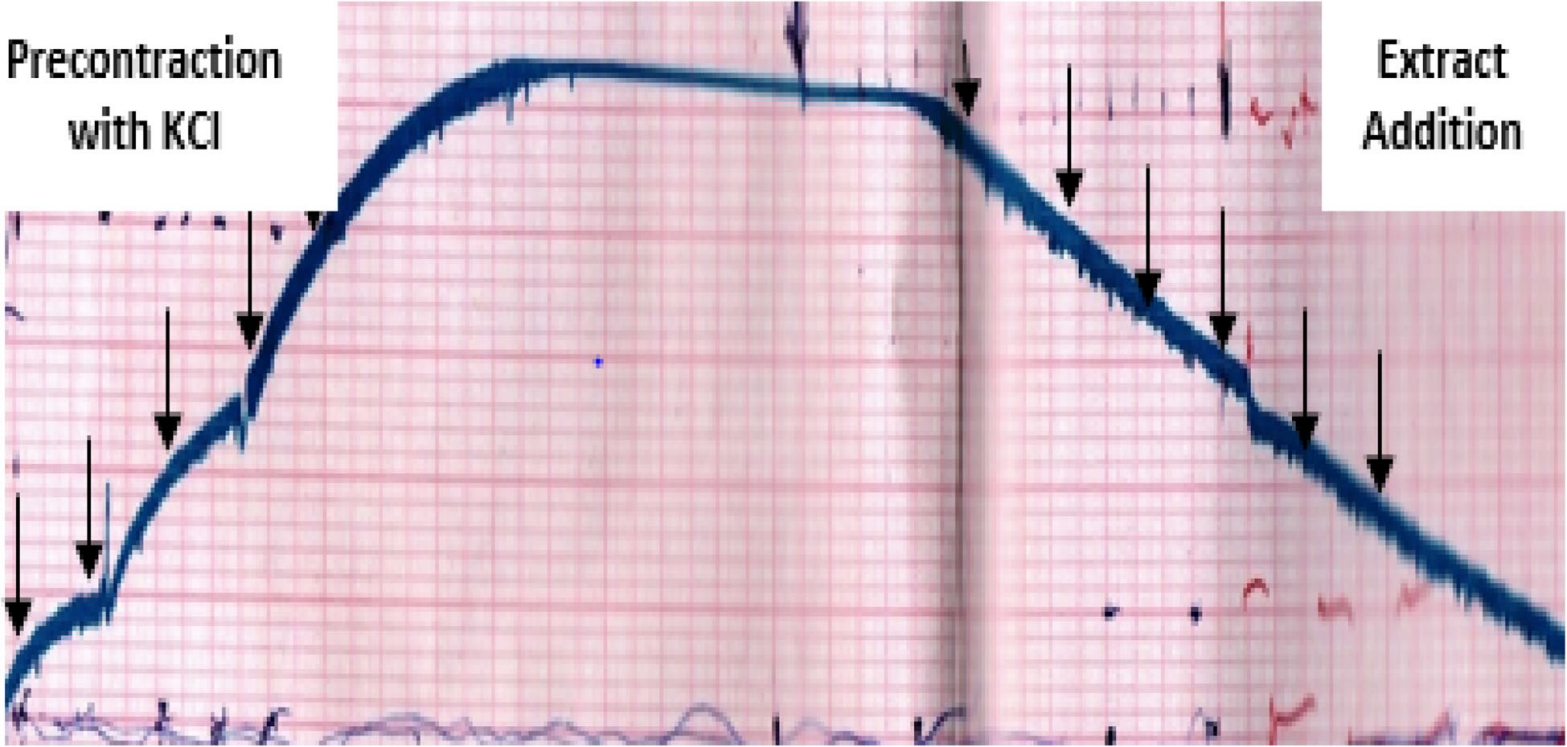
Typical tracing showing relaxant effect of 80% methanol leaf extract of *Otostegia integrifolia* on high K^+^ (80 mM)-induced contraction on isolated aorta strips of rat

Initial assessment was performed on resting baseline aortic strips to see whether the extract possessed vasoconstrictor activity and found to be devoid of the said activity. Afterwards, the effect of the extract was assessed following high-K^+^ (80 mM) induced contraction (Fig. 5). K^+^ induced a contraction with pEC50 of 1.61 ± 0.004 and the extract demonstrated a concentration-dependent vasodepressor activity (EC_50_ of 63.5 μg/ml), with a maximum relaxation attained at a cumulative concentration of 318.75 μg/ml (Table 4).

**Table 4:** Vasorelaxant effect of *Otostegia integrifolia* extract on rat thoracic aorta pre-contracted with 80 mM KCl

Concentration (μg/mL)	% Relaxation by the extract in KCl precontracted aorta
0.00	0.00 ± 0.00
6.25	1.25 ± 0.55
12.5	6.5 ± 1.98
25	21.0 ± 2.54***
50	44.5 ± 2.61***
100	76.3 ± 2.41***
125	100.00 ± 00***

***p < 0.001 (*n* = 10. Results are expressed as mean percentage contraction or relaxation ± SEM)

At lower concentrations, particularly 6.25 μg/ml, and 12.5 μg/ml, the observed percentage relaxation of tissues contracted with K^+^ was not statistically significant. With subsequent increment in concentration, beginning at a concentration of 25 μg/ml, however, significant (*p* < 0.001) percentage relaxation was recorded.

Relaxation began within a couple of seconds and extended to some more minutes with addition of the extract into the bath. This may indicate that the vasodepressor effect of the test substance followed both time and concentration-dependent pattern. After subsequent washout, the tissue regained its activity, possibly suggesting that the effect of the extract is reversible (Table 4).

#### Possible mechanism of vasorelaxation

To assess the possible underlying mechanism (s) for the observed vasodilation, the isolated thoracic aorta was subjected to different manipulations, including, among others, using agonists and antagonists/inhibitors of different receptors/enzymes thought to be involved in the relaxation mechanism. Incubating the tissue with inhibitors/blockers of signaling molecules acting through the endothelium, including acetylcholine (Atropine) [27], prostacyclin (PGI_2_) (Indomethacin) [30], nitric oxide synthase (L-NAME), and histamine (diphenhydramine) [31–33] did not alter the extract induced relaxation. The endothelium is known to produce vasodilation via NO, prostacyclin and endothelium derived hyperpolarizing factor (EDHF). As the aforementioned manipulation do not include EDHF, studies were conducted on a denuded aorta. Denuding the aorta, however, did not alter the relaxation, ruling out the role of the endothelium. Once the endothelium was ruled out, the other possibilities left were potassium- and calcium-based mechanisms. Pretreatment with glibenclamide, an ATP-dependent K^+^ channels blocker [34, 35] did not also affect the vasodepressor activity, ruling out the role of K^+^ channels in the observed effect. Intracellular calcium is known to be derived from subcellular and extracellular sources. Incubation with phenylephrine, an α-1 receptor agonist causing the release of Ca^2+^ from intracellular stores via IP_3_ [34, 36], did not also have effect on the extract.

**Fig. 6:**
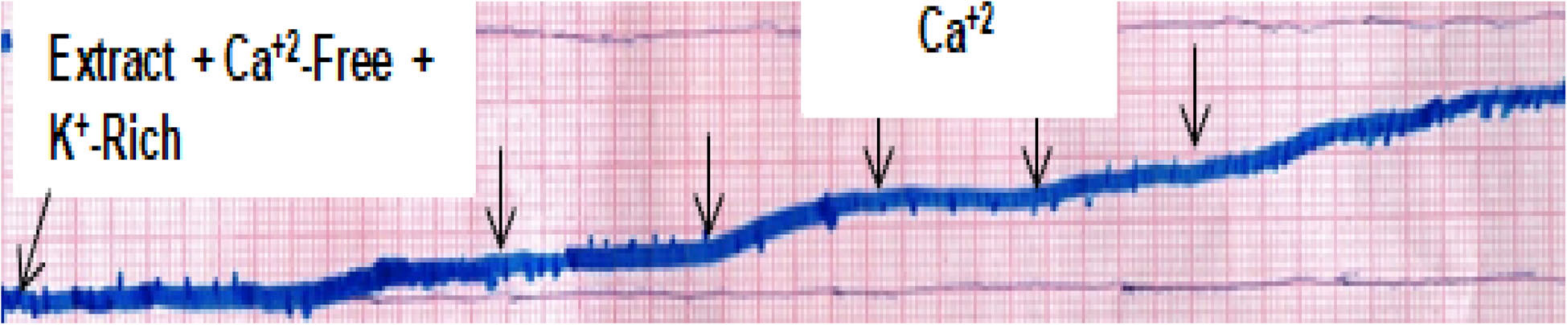
Typical tracing showing 80% methanol leaf extract of *Otostegia integrifolia* partially prevents contraction of isolated rat aortic strip upon gradual increment in concentration of Ca^++^

**Fig. 7:**
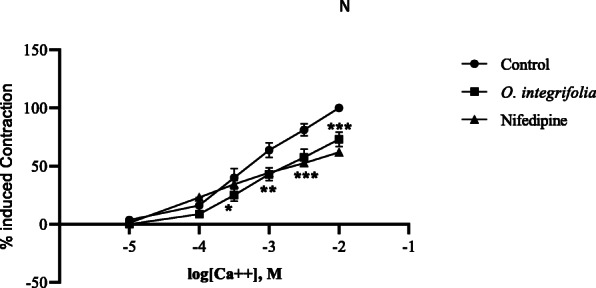
Concentration-response curve of Ca^++^ in the absence and presence of 80% methanol leaf extract of *Otostegia integrifolia* and nifedipine: concentration used in the isolated rat aortic strip preparations was the highest concentration of the crude extract (318.75 ug/mL,) and 0.03 mM nifedipine. (*n* = 4. *p < 0.05, **p < 0.01 and ***p < 0.001 compared to the respective concentration values in the Ca^++^ control curve)

Following these observations, the role of calcium channels was investigated on a denuded aorta. For this purpose, a CRC was constructed in a Ca^2+^-free K^+^ rich medium through continuously raising Ca^2+^ concentration in the bath so that a gradual increase in tension could be induced. Pretreatment of the denuded aorta with a cumulative maximum concentration (318.75 μg/mL) of the extract that resulted in maximum relaxant effect for 60 min shifted the CRC (*p* < 0.05, *p* < 0.01 and *p* < 0.001) to the right with suppression of the maximum effect (Figs. 6 and 7). The extract as well as nifedipine (0.3 mM), a CCB, prevented the Ca^2+^ induced maximum contraction of the aortic strips by 26.9 and 38.1%, respectively, from the control value (*p* < 0.001). Furthermore, to make sure that whether the mechanism is only mediated with calcium channel pathway, the tissue was also pre-incubated with nifedipine, but the extract did not show any significant relaxation.

## Discussion

In the present study, the antihypertensive activity of *O. integrifolia,* a folkloric herbal medicine, was evaluated in fructose-induced hypertension. The study showed that subacute administration of 80% methanol leaf extract significantly reduced BP in fructose-induced hypertensive rats. Fructose is known to cause hypertension through salt retention, endothelial dysfunction, hyperinsulinemia, upregulation of the RAAS. [7]. Although fructose was able to cause a significantly higher BP at all time points compared to NCR, consistent increase in BP was not obtained after Day 5, suggesting that compensatory mechanisms might have been activated, with continuous ingestion, that had attenuated the further increase in BP. It is thus apparent from the experiment that the hypertension was relatively mild, as fructose-induced metabolic derangements had to overwhelm the regulatory pathways [37].

Traditionally, the leaves were boiled with water to facilitate the extraction process [13, 20]. However, 80% methanol was used in the present experiment, as it is generally considered a universal solvent that can extract polar and semi-polar constituents and the intermolecular forces that govern the extraction process in both solvent systems are hydrogen bonds. In addition, 80% methanol extract of the plant is demonstrated to exhibit antimalarial [16] as well as anti-diabetic [38] activity.

The extract showed a consistent reduction in BP at both the middle (OI500) and higher (OI1000) doses, with no significant difference between the two doses, possibly suggesting that 500 mg/kg is the maximum dose and further escalation of the dose might not be accompanied by an increase in response [39]. OI250 could be considered as the minimum effective dose, as significant reduction was obtained on days 5 & 15, though the reduction on day 10 failed to reach statistical significance. This could possibly be due to small sample size and greater variation among individual values, which precluded statistical significance.

Although the extract, particularly OI500, showed a statistically significant reduction in all BP parameters, a relatively strong effect was observed on DBP than SBP. Based on this observation and the fact that the extract's antihypertensive effect was mediated by vasorelaxation, it is plausible to assume that the extract might have a strong relaxant effect on the venous than the arterial bed. As a result, venous return to the heart would be decreased and preload is preferentially lowered than afterload [40].

The ex vivo study was conducted to shed light on the possible mechanism of action for the antihypertensive activity of the extract. Exposure of smooth muscles of aortic strips to high concentration of K^+^ is known to cause contraction, probably mediated by Ca^2+^ mobilization [41]. The extract did not affect contraction until it reaches a threshold concentration, 25 μg/mL, after which it showed a concentration-dependent relaxation. This observation is consistent with the in vivo study, as increasing the dose of the extract also produced an increase in response. This similarity in response, in both studies, could probably point to the fact that the observed in vivo antihypertensive effect of the extract is mediated through vasodilation of blood vessels.

It is well known that the mechanisms by which natural products and plant extracts induce vasorelaxation are linked to the NO system, eicosanoid system and K+ channel functions [42–44]. Indeed, endothelial cells elaborate several vasoactive mediators that play an important role in vascular homeostasis by modulating vasomotor tone and multiple K^+^ channels (K_Ca_ or K_ATP_), which are also present in most vascular smooth muscle cells and play unique roles in regulating vascular tone [45]. However, denuding the aorta as well as preincubation of the aortal strip with inhibitors of the endothelial system and K^+^ channels did not affect the response, suggesting the relaxation involve neither the PI3K/Akt-eNOS-NO-cGMP nor the prostaglandin signaling pathway.

Plant extracts are also shown to induce vasorelaxation through blockade of voltage-gated calcium channels [46]. Pretreatment of the tissue with the extract inhibited calcium-induced contraction and resulted in rightward non-parallel shift of Ca^2+^-CRCs. This shift was similar to the one produced by nifedepine, a dihydropyridine CCB, albeit the effect was smaller than the comparator, suggesting that the effect may probably be mediated through blocking of the calcium channels.

Previous studies conducted on the hydroalcholic extract of a related species, *O. persica,* showed that the plant is endowed with BP lowering activity, and terpenes and flavonoids were implicated for the observed effect [47]. Phytochemical investigations of *O. integrifolia* indicated that it contains terpenes, phenolic compounds, saponins, and flavonoids [16]. The present study also confirmed that the plant is rich in total phenols and flavonoids. For instance, flavonoids in particular quercetin, is known to bind to zinc, which is found in the active site of angiotensin converting enzyme, thereby inhibiting the RAAS. Quercetin is also shown to downregulate endothelin-1 expression and to induce vasorelaxation in endothelium denuded aortic tissue through blockade of voltage gated calcium channels [48–50]. Similarly, saponins extracted from soybean are shown to reduce BP through inhibition of renin, thereby attenuating the RAAS pathway [51]. Terpenoids isolated from various plants were also shown to reduce BP mainly via inhibition of the L-type calcium channels [52]. Although the BP lowering effect of these constituents and their role in inhibiting fructose induced hypertension was not investigated, the data collectively suggest that the plant's antihypertensive activity might be due to the individual or synergistic activity of the phytochemicals determined in the present study.

## Conclusions

The result from the in-vivo experiment demonstrated that the extract significantly lowered SBP and DBP as well as MAP, indicating the extract’s promising antihypertensive activity with the method employed in this experiment. The ex vivo experiments also indicated that the extract produced a concentration-dependent vasodepressor activity probably mediated through blockade of L-type calcium channels. The overall investigation upholds the traditional claim of the plant for its antihypertensive property.

## Supplementary Information

**Additional file 1: Figure S1.** Calibration curve used to calculate phenolic compounds. **Figure S2.** Calibration curve used to calculate flavonoid content. **Figure S3.** Calibration curve used to calculate EC50. **Figure S4.** Typical tracing showing relaxant effect of 80% methanol leaf extract of *Otostegia integrifolia* in the presence of inhibitor and contracted with high K+ (80 mM) on isolated aorta strips of rat.

## Data Availability

The information supporting the conclusions of this article is included in the article and its supplementary information file.

## Abbreviations

BP: Blood pressure
CAP: Captopril
CCB: Calcium channel blocker
cGMP: Cyclic guanosine monophosphate
CRC: Concentration response curve
DBP: Diastolic blood pressure
DF66: D-fructose 66%
EDHF: Endothelial derived hyperpolarizing factor
IP_3_: Inositol triphosphate
L-NAME: N(ω)-nitro-L-arginine methyl ester
MAP: Mean arterial pressure
NCR: Normal control rat
NO: Nitric oxide
OI: *Otostegia integrifolia*
PP: Pulse pressure
RAAS: Renin angiotensin aldosterone system
SBP: Systolic blood pressure
