# Supplementary material for: Methanol (80%) leaf extract of Otostegia integrifolia Benth (Lamiaceae) lowers blood pressure in rats through interference with calcium conductance
Source: BMC Complement Med Ther. 2021 Feb 4;21:49. doi: 10.1186/s12906-021-03222-4 (PMC7863373; doi:10.1186/s12906-021-03222-4)
Supplement: Supplementary file 1 — Additional file 1: Figure S1. Calibration curve used to calculate phenolic compounds. Figure S2. Calibration curve used to calculate flavonoid content. Figure S3. Calibration curve used to calculate EC50. Figure S4. Typical tracing showing relaxant effect of 80% methanol leaf extract of Otostegia integrifolia in the presence of inhibitor and contracted with high K+ (80 mM) on isolated aorta strips of rat. [file 12906_2021_3222_MOESM1_ESM.docx]

**Supplement**

**Figure 1S:** Calibration curve used to calculate phenolic compounds

**Figure 2S:** Calibration curve used to calculate flavonoid content

**Figure 3S:** Calibration curve used to calculate EC50


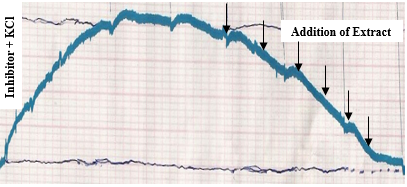


**Figure 4S:** Typical tracing showing relaxant effect of 80% methanol leaf extract of *Otostegia integrifolia* in the presence of inhibitor and contracted with high K+ (80 mM) on isolated aorta strips of rat.
